# Functional activity within the frontal eye fields, posterior parietal cortex, and cerebellar vermis significantly correlates to symmetrical vergence peak velocity: an ROI-based, fMRI study of vergence training

**DOI:** 10.3389/fnint.2014.00050

**Published:** 2014-06-17

**Authors:** Tara L. Alvarez, Raj Jaswal, Suril Gohel, Bharat B. Biswal

**Affiliations:** Department of Biomedical Engineering, New Jersey Institute of TechnologyNewark, NJ, USA

**Keywords:** vergence, frontal eye fields, posterior parietal cortex, cerebellar vermis, vision therapy, vergence training, convergence insufficiency, Convergence Insufficiency Symptom Survey

## Abstract

Convergence insufficiency (CI) is a prevalent binocular vision disorder with symptoms that include double/blurred vision, eyestrain, and headaches when engaged in reading or other near work. Randomized clinical trials support that Office-Based Vergence and Accommodative Therapy with home reinforcement leads to a sustained reduction in patient symptoms. However, the underlying neurophysiological basis for treatment is unknown. Functional activity and vergence eye movements were quantified from seven binocularly normal controls (BNC) and four CI patients before and after 18 h of vergence training. An fMRI conventional block design of sustained fixation vs. vergence eye movements stimulated activity in the frontal eye fields (FEF), the posterior parietal cortex (PPC), and the cerebellar vermis (CV). Comparing the CI patients' baseline measurements to the post-vergence training data sets with a paired *t*-test revealed the following: (1) the percent change in the BOLD signal in the FEF, PPC, and CV significantly increased (*p* < 0.02), (2) the peak velocity from 4° symmetrical convergence step responses increased (*p* < 0.01) and (3) patient symptoms assessed using the CI Symptom Survey (CISS) improved (*p* < 0.05). CI patient measurements after vergence training were more similar to levels observed within BNC. A regression analysis revealed the peak velocity from BNC and CI subjects before and after vergence training was significantly correlated to the percent BOLD signal change within the FEF, PPC, and CV (*r* = 0.6; *p* < 0.05). Results have clinical implications for understanding the behavioral and neurophysiological changes after vergence training in patients with CI, which may lead to the sustained reduction in visual symptoms.

## Introduction

Throughout the day, the visual system mediates vergence eye movements to acquire visual information located at different spatial depths from the retina using the medial and lateral recti muscles. The inward rotation of the eyes is known as convergence. Convergence insufficiency (CI), a prevalent binocular vision disorder in adults (Porcar and Martinez-Palomera, 1997) and children (Rouse et al., 1999), is typically characterized by reduced fusional convergence amplitude, receded near point of convergence (NPC), greater exophoria at near than at far and visual symptoms. The visual symptoms commonly experienced by CI patients include the following: double/blurred vision, eyestrain, and headaches when engaged in reading or other near work, thus interfering with activities of daily living (Scheiman et al., 2011; Lee et al., 2014). Exophoria is the outward deviation of the eye when binocular fusion is disrupted (i.e., one eye is occluded while the other eye is fixating on a target). Scheiman and others hypothesize that CI patients are symptomatic because of the excessive convergence needed to compensate for a larger exophoria at near (40 cm) compared to far (6 m) (Cooper et al., 1998; Scheiman and Wick, 2008; Scheiman et al., 2011; Cooper and Jamal, 2012). Double vision is a common symptom of CI patients which could be explained by a reduced speed of convergence eye movements. Thus, their convergence responses require more time to attain fusion leading to double vision. Several investigations also report that vergence peak velocities elicited from abrupt changes in disparity are reduced in those with CI compared to binocularly normal controls (BNC) and improves to levels more similar to controls after repetitive vergence training (Van Leeuwen et al., 1999; Alvarez et al., 2010b; Thiagarajan et al., 2011; Alvarez and Kim, 2013).

Randomized clinical trials report that Office-Based Vergence and Accommodative Therapy with home reinforcement (OBVAT) reduces the visual symptoms in CI patients (Scheiman et al., 2009, 2011). The reduction of visual symptoms is sustained 1 year post-therapy in most subjects (CITT, 2009). Clinicians commonly prescribe vergence training (also known as vision therapy, vision rehabilitation, or orthoptic exercises) to reduce visual symptoms (Scheiman et al., 2011; Cooper and Jamal, 2012). However, the true neural mechanism by which vergence training leads to a reduction in visual symptoms is currently unknown (Scheiman et al., 2011).

There are many single cell and lesion studies on primates, as well as human case reports and fMRI studies that form the basis of our understanding of the neural substrates used to mediate a convergence response. Studies on primates report that the convergence circuit does involve cortical areas within the posterior parietal cortex (PPC) where cells have been identified that have a preferred direction for targets closer or farther away (Gnadt and Mays, 1995; Sakata et al., 1999; Taira et al., 2000; Genovesio and Ferraina, 2004). Single cell recordings from primates reveal a distinct area within the frontal eye fields (FEF) that is allocated for step convergence responses and is located more anterior compared to the cells responsible for generating saccadic responses (Gamlin and Yoon, 2000). Our team reports differentiation within FEF between saccadic and step convergence responses using fMRI (Alvarez et al., 2010a; Alkan et al., 2011a). Other investigators support that smooth convergence tracking is also encoded within FEF using single cell recording from primates (Kurkin et al., 2003; Akao et al., 2005) and using fMRI (Petit and Haxby, 1999). Many studies support the cerebellum is active during convergence responses (Miles et al., 1980; Gamlin and Clarke, 1995; Zhang and Gamlin, 1998; Gamlin, 2002; Takagi et al., 2003; Nitta et al., 2008a,b). In addition, lesions to the cerebellar vermis (CV) VI/VII in primates (Takagi et al., 2003) and humans (Sander et al., 2009) result in a convergence dysfunction. Many single cell studies also show neural activity within the midbrain (Mays et al., 1986; Zhang et al., 1991, 1992). In summary, the PPC, FEF, cerebellum, and midbrain are utilized to generate a convergence response.

Functional imaging investigations non-invasively quantify the metabolic demand generated through an experimental task by studying the blood oxygen level dependent (BOLD) response. For this study, we will measure the paramagnetic properties of blood during sustained fixation compared to active vergence eye tracking. The portions of the brain that are more metabolically active during vergence eye tracking will be assumed to be responsible for generating a vergence response. Functional imaging signals from the cerebrum and cerebellum are easier to obtain compared to signals within the brainstem. The brainstem is more susceptible to breathing and swallowing motion artifacts compared the cerebrum and cerebellum and hence is beyond the scope of this present study. Vergence training leads to a sustained reduction in symptoms suggesting neuroplasticity. Based upon the aforementioned studies about the neurophysiology of the vergence circuit, this investigation analyzed the PPC, FEF, and CV before and after vergence training in patients with CI compared to BNC. BNC did not participate in vergence training since they did not have visual symptoms. This research takes a critical step in understanding the neural basis of how vergence training leads to a sustained reduction of vision symptoms in patients with CI. Such knowledge may ultimately lead to new vergence training protocols to further improve vision function.

This study investigated convergence responses and functional activity of the vergence neural substrates before and after repetitive vergence training in symptomatic CI patients compared to BNC subjects. The following hypotheses were tested. First, reduced convergence peak velocity elicited from symmetrical convergence step stimuli and reduced functional activity within the FEF, PPC, and CV would be observed in those with CI before repetitive vergence training compared to BNC subjects. Second, after repetitive vergence training, the peak velocity of symmetrical convergence step responses would increase along with the percent change in functional activity of the vergence neural substrates from CI patients compared to their baseline measurements. Third, the peak velocity of convergence responses would significantly correlate to the functional activity of the FEF, PPC, and CV neural substrates quantified as the BOLD percent signal change.

## Materials and methods

### Subjects and vision parameters

Four CI (four females) and seven BNC subjects (three females) participated in this study. CI was diagnosed by an optometrist using methods described in our prior study, which included a receded NPC and reduced positive fusional amplitudes (Alvarez et al., 2010b). The diagnosis criteria comply with conventional clinical methods (Cooper et al., 1998). At the beginning of the study, the CI subjects had the following parameters denoted as the average with one standard deviation: near point convergence of 13.4 ± 5.6 cm, positive [base out (BO)] vergence amplitude of 14 ± 4.5Δ, and a dissociated near (measured at 40 cm from midline) phoria of 9.0 ± 1.4Δ exophoria. NPC was assessed by measuring the distance from the orbit to the location where a high acuity target was perceived as diplopic along the subject's midline (Von Noorden and Campos, 2002). Stereopsis was assessed by the Randot Stereopsis Test (Bernell Corp., South Bend, IN, USA). Normal binocular vision was defined as having a NPC of less than 8 cm. The inclusion criteria were as follows: normal stereopsis, no ocular surgeries and corrected to normal acuity. In addition, if an eye spectacle prescription was required then subjects who required a prescription greater than 2D or less than -3D were excluded to reduce potential confounding variables. All subjects had a stereopsis of ≤50 s of arc. Three of the CI and five of the BNC did not need spectacles to read clearly at near. All subjects had no history of brain disorders and were between the ages of 18 and 35 years. All subjects signed written informed consent forms approved by the University of Medicine and Dentistry of New Jersey (UMDNJ) and New Jersey Institute of Technology (NJIT) Institution Review Boards (IRB) in accordance with the Declaration of Helsinki.

### Convergence insufficiency subject symptoms

Symptoms were quantified using the Convergence Insufficiency Symptom Survey (CISS), which contains 15 questions (CITT, 2008). All questions were in regard to the subject's ability to read or perform near work. Each symptom was scored between zero and four where zero represents the symptom never occurs and four represents the symptom occurs very often. A prior investigation compared visual symptoms to the clinical diagnosis of CI defined as an exophoria at near at least 4Δ greater than at far, failure of Sheard's criteria or a minimum normal positive fusional vergence (break < 15Δ), and a receded (≥6 cm) NPC (Rouse et al., 2004). The responses of CISS were summed where a score of 21 or higher had a sensitivity of 98% and specificity of 87% using the aforementioned diagnostic criteria of CI in young adults between the ages of 18 and 35 years of age (Rouse et al., 2004). Hence, the CISS symptom survey was used within this present investigation to assess visual symptoms of the CI and BNC subjects who participated in this study.

### Overall experimental protocol goals

The CI subjects participated in 18 h of vergence training as described below. The CI data were compared to BNC data where BNC subjects did not participate in vergence training since they did not have visual symptoms. The primary measurements compared were the peak velocity from convergence step responses and the percent change in the BOLD signal within the FEF, PPC, and CV. Secondary measurements included the NPC, positive fusional range, near dissociated phoria and the CISS score. A group level analysis compared the following: (1) the BNC data to the CI baseline data and (2) the CI pre and post-vergence training measurements.

### Vergence training protocol for convergence insufficiency subjects

Repetitive vergence training was designed to provoke changes in the neural substrates (FEF, PPC, and CV) that stimulate vergence ocular motor responses. The CI subjects participated in a total of 18 h of vergence training, 6 h at home, and 12 h in the laboratory. Home training was monitored by having each CI subject record the amount of time spent on training and entailed two 10-min sessions (morning and evening) 3 days per week for 6 weeks. Laboratory training was composed of 1-h sessions, twice per week for 6 weeks. Within a single day, a subject participated in either laboratory or home training but not both.

For the laboratory step training, 2, 4, and 6° disparity convergence steps and 4° disparity divergence steps within the range of a 2° vergence angle to 8° vergence angle were presented after a randomized delay of 0.5–2.0 s. The randomized delay reduces anticipatory cues that are known to alter the latency and peak velocity of vergence responses (Alvarez et al., 2002, 2005, 2010a; Kumar et al., 2002a,b). The ramp training consisted of 1 and 2°/s ramps starting at an initial vergence angle of 2° producing a convergence ramp response to the vergence angle of 8° and then stimulating a divergence ramp response to a vergence angle of 2°. The laboratory and home training consisted of step and ramp stimuli similar to methods used clinically (Griffin, 1988; Scheiman and Wick, 2008).

### Eye movement acquisition and analysis

Eye movements were recorded using a Skalar Iris (model 6500, Delft, Netherlands) infrared (λ = 950 nm) limbus tracking system. The manufacturer reports that the linear range of the system was ± 25° where all responses of this study were within the linear range of the device. Prior research confirms a high degree of linearity, within 3% between 5° horizontally for this system (Horng et al., 1998). A 12-bit acquisition hardware card (National Instruments 6024 E series, Austin, TX, USA) digitized the individual left-eye and right-eye movements with a sampling rate of 200 Hz. The visual stimuli utilized green light emitting diodes (LEDs) (Stanley model MU07 part 5101, London, OH, USA) 2 mm wide by 25 mm in height with a wavelength of 555 nm. Subjects were situated in a head and chin rest assembly to reduce any influence from the vestibular system (Khojasteh and Galiana, 2007). The subject initiated each experimental trial by pressing a button, which allowed the subject to blink between experimental trials. Potential subject fatigue was also reduced by allowing the subject to initiate the experimental trial (Yuan and Semmlow, 2000).

A custom MATLAB™ (Waltham, MA, USA) program was used for all eye movement analyses. Left-eye and right-eye movement data were converted from voltage values into degrees using the individual calibration data. Eye movements were calibrated using 2, 4, 6, and 8° vergence angles. Vergence was calculated as the difference between the right-eye and the left-eye position to yield a net vergence response. Convergence responses were plotted as positive. Blinks were easily identified based upon manual inspection of the left-eye and right-eye movement response. Responses with blinks at any point during the movement were omitted (up to 2.1% of the data depending upon the subject). Only convergence responses were analyzed since convergence responses were reported to have reduced peak velocities in CI subjects compared to BNC (Alvarez et al., 2010b).

Peak velocity generated from a 4° symmetrical convergence step stimulus was a primary measurement within this study. Velocity was computed by taking the derivative of the position response using a two-point central difference algorithm (Bahill et al., 1982). Each individual left-eye and right-eye convergence movement response was manually inspected for the presence of a saccade, which was easily identified because saccade velocities are an order of magnitude greater than vergence. A phase plot (vergence velocity as a function of vergence amplitude) for the left-eye and the right-eye movement was used to determine whether the saccades obscured the peak velocity of the vergence response to the symmetrical stimulus. Only when saccades obstructed the convergence peak velocity was the response omitted from the peak velocity analysis, which occurred in less than 10% of the responses depending on the subject as shown in our prior investigations (Alvarez et al., 1998; Lee et al., 2008; Kim and Alvarez, 2012). The peak velocity of the combined vergence response was quantified as the maximum value.

### Imaging instrumentation and acquisition

A 3-Tesla Siemens Allegra Magnetron MRI Scanner with a standard single channel head coil (Erlangen, Germany) was used to perform the fMRI scans during the experimental tasks. The fMRI imaging parameters used during the acquisition included: repetition (*TR*) = 2000 ms, echo time (*TE*) = 27 ms, matrix size = 64 × 64, field of view (FOV) = 220 mm, and flip angle = 90°. A total of 32 slices were collected (axial orientation) with a slice thickness of 5 mm. The voxel resolution was 3.4 × 3.4 × 5.0 mm. High resolution anatomical volumes acquired using a magnetization-prepared rapid acquisition with gradient echo (MPRAGE) were collected after all functional tasks. The MPRAGE imaging parameters included the following attributes: *TR* = 7.2 ms, *TE* = 4.38 ms, *T*1 = 900 ms, flip angle = 8°, matrix size = 256 × 256 with a total of 80 acquired slices. The voxel resolution was 0.9 × 0.9 × 2.0 mm. Subjects were instructed to limit head motion and foam padding was used to facilitate the restriction of physical movement. All subjects were positioned supine on the gantry of the scanner with their heads situated along the midline of the coil.

### Functional MRI visual stimulus experimental design

**Figure 1:**
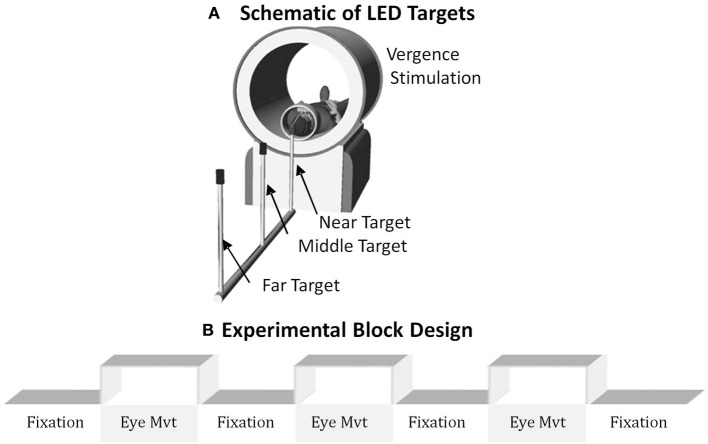
**(A)** Schematic of the LED targets showing visual stimuli. **(B)** The experimental block design was composed of sustained fixation (denoted as Fixation) and vergence eye movements (denoted as Eye Mvt). This experimental block design modulated functional activity of the BOLD signal within the vergence neural substrates.

The visual stimulus (see Figure 1A) was carefully aligned with the subject's midline to stimulate symmetrical vergence eye movements to test the hypotheses of this study. Subjects could see the targets with the aid of a mirror. Visual stimuli were a set of non-ferrous LED targets that formed a line 5 cm in height by 2 mm in width secured with polyvinyl chloride (PVC) tubing. The LED stimulus targets were located at the following three full vergence angle demands: 2, 3, and 4°. The target positions were chosen because smaller vergence movements have been shown to elicit fewer saccadic responses compared to larger vergence movements (Coubard and Kapoula, 2008; Semmlow et al., 2008, 2009; Chen et al., 2010).

The experiment utilized a conventional block design of sustained fixation for the “off” stimulus compared to vergence eye movements for the “on” stimulus as shown in Figure 1B. Anticipatory cues are known to decrease the latency and increase peak velocity of convergence responses (Alvarez et al., 2002, 2005, 2010a; Kumar et al., 2002a,b). Hence, to reduce anticipatory or predictive cues, this experiment utilized a series of vergence eye movements where each target was illuminated for a random duration between 3 and 5 s. LED targets were never simultaneously illuminated. The eye movement sequence illuminated one of the following three stimuli: the near (4°), middle (3°), or far (2°) target where the subject could not anticipate when the next target would illuminate or which of the three targets would be illuminated. Each phase lasted 20 s and was repeated for 3.5 cycles. Hence, the total experiment time was 2 min 20 s. The experiment was repeated three times per subject.

### Imaging analysis

#### Image preprocessing

The AFNI (Cox, 1996) and FSL (Jenkinson and Smith, 2001; Jenkinson et al., 2002) software suites were used to process and analyze the raw data retrieved from the MRI scanner. The first five images of each trial dataset were removed to mitigate the effect of transient scanner artifact (Biswal et al., 2010).

The AFNI motion correction utilizes the application of a six-parameter, rigid-body, least-squares alignment routine. Three parameters calculate the amount (mm) of movement within each plane (anterior to posterior, right to left, and inferior to superior) and three parameters calculate the amount of rotation (°) between planes (yaw, pitch, and roll). These six motion regressors are used within the linear regression model to minimize motion effects of the acquired BOLD signal. A detailed motion analysis of all subjects using a frame displacement method which calculates the absolute value of movement was conducted (Satterthwaite et al., 2013). The average frame displacements with one standard deviation for the degree of rotation were 0.18 ± 0.07°, 0.16 ± 0.09°, and 0.20 ± 0.08° for yaw, pitch, and roll, respectively. The average frame displacement analyzing all subjects within each plane, with one standard deviation, were 0.36 ± 0.13 mm, 0.42 ± 0.11 mm, and 0.37 ± 0.08 mm for the anterior to posterior, left to right, and inferior to superior planes, respectively. No significant differences in motion artifacts were observed between the post and pre-vergence training data sets of the CI patients assessed using a paired *t*-test (*p* > 0.9). No significant difference in head motion was observed between the two groups (CI compared to BNC) (*p* > 0.9). Hence, head motion was not considered problematic within this dataset.

The CompCor data-driven method was used to further reduce effects of noises in the BOLD signal, as described below (Behzadi et al., 2007). FSL's BET (Brain Extraction Tool) (Smith, 2002) function removed non-brain tissue from the anatomical image dataset. FSLs FAST (FMRIBs Automated Segmentation Tool) (Zhang et al., 2001) stratifies the skull-stripped anatomical dataset into three different segments. The whole brain probability maps of CSF, WM, or gray matter (GM) were derived. The segmented anatomical CSF and WM probability images were transformed into functional space using FSL's FLIRT function (Beckmann and Smith, 2004, 2005). To create CSF and WM regressors, all voxels of the CSF and WM probability images were first thresholded using levels of 99 and 97% probability, respectively. Time-series from all the voxels surviving the threshold were extracted. The probability levels of this study are more conservative compared to those used previously, which used a threshold level of 80% (Biswal et al., 2010). Then, the first five principle components relating to CSF and WM time-series were calculated. FSL's FEAT command was used to perform the voxel-wise linear regression analysis on all datasets using the 16 aforementioned regressors (six motion parameters, five principle components of CSF, and five principle components of WM). The residuals of the regressed datasets (removal of the 16 artifacts) were then filtered in AFNI using a band pass filter [full width at half maximum (FWHM) Gaussian filter with cut off frequencies of 0.01 and 0.15 Hz]. The band pass filter was used to remove DC offset and high frequency signals that were probably not neuro-physiological in nature. Following band-pass filtering, a general linear model (GLM) analysis was performed to derive functionally active regions during the task.

#### General linear model

A GLM using a reference time series representation of the block design experimental stimulus convolved with the hemodynamic response function (HRF) was used. Correlation maps were created using a threshold of *r* ≥ 0.4 (*p* < 0.05) to show active brain regions. Mask identification was facilitated by observing the active brain regions coupled with the anatomical locations described above for the FEF, PPC, and CV. Broca's region was the control region of interest (ROI) and was identified strictly using anatomical markers. Since the datasets were not transformed into a standardized space such as the Montreal Neurological Institute (MNI) space, some variance was also observed for the mask of Broca's region. Broca's region served as a control ROI (unrelated to the hypotheses of this study). Language was not manipulated within the experimental protocol. Prior investigations show Broca's region was stimulated during experiments that study language (Geschwind, 1970; Kim et al., 1997) but is not stimulated within vergence eye movement experiments (Alkan et al., 2011a,b).

#### Cortical and subcortical regions of interest (ROIs) within the fMRI experiment

The ROIs were defined using anatomical markers coupled with a model-driven method to identify functional activity near the anatomical markers. Neurophysiology studies on primates support the following ROIs are involved in vergence eye movements: FEF, PPC, and CV (Gamlin et al., 1996; Gamlin and Yoon, 2000). This experiment sought to stimulate the cortical and cerebellar regions required to mediate vergence eye movements.

**Figure 2:**
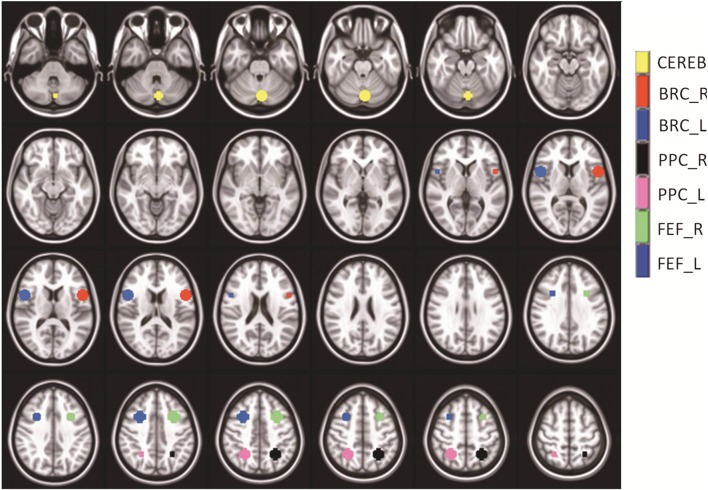
**Series of axial images showing the average masks with one standard deviation used within the analysis**. The cerebellar vermis (yellow), Broca-Right (red), Broca-Left (dark blue), Posterior Parietal Cortex–Right (black), Posterior Parietal Cortex–Left (pink), Frontal Eye Field-Right (green), and Frontal Eye Field–Left (medium blue) are shown. The masks did not overlap. Broca's Region served as a control ROI to study the variability within a non-stimulated ROI.

The following ROIs were drawn in native space using anatomical markers and functional activity derived using a GLM: FEF, PPC, and CV. The bilateral FEFs were defined as the area within the intersection between the precentral sulcus and superior frontal sulcus. The PPC was around the intraparietal sulcus as shown in Figure 2. The CV regions VI and VII were defined on the mid-sagittal plane. Broca's region served as a control ROI because it was not stimulated in prior fMRI vergence studies (Alvarez et al., 2010a; Alkan et al., 2011a,b). The mask for Broca's region was created using only anatomical markers that are defined near the inferior frontal gyrus anterior to the motor strip as shown in Figure 2. Figure 2 depicts the ROIs within a series of axial slices. The average with one standard deviation for the masks studied (measured in mm^3^) were 880 ± 118, 901 ± 126, 1430 ± 275, 1194 ± 382, 1006 ± 131, 499 ± 58, 541 ± 47 for the FEF-L, FEF-R, PPC-L, PPC-R, CV, Broca-L, and Broca-R, respectively. The average and one standard deviation of each ROI are shown in Figure 2 using an MNI template. As Figure 2 shows, none of the masks overlap to avoid any partial volume effects. The centroid of the mask listed as left (positive) or right (negative), anterior (positive) or posterior (negative), and superior (positive) or inferior (negative) are (30, 12, 42), (−30, 12, 42), (52, 10, 12), (−52, 10, 12), (−2, −74, −28), (26, −54, 48), and (−26, −54, and 48) for the FEF-L, FEF-R, PPC-L, PPC-R, CV, Broca-L, and Broca-R, respectively.

#### Analysis of percent change of BOLD signal to quantify functional activity

All data were kept in native space (i.e., data were not transformed into Talairach and Tournoux or MNI space) to avoid any warping artifacts. The time series located within the vicinity of the anatomical markers, which had a Pearson correlation coefficient of *r* ≥ 0.4 (*p* < 0.05) with the hemodynamic model described above, were pooled. While the percent signal change will be threshold dependent, this study is longitudinal comparing the data after vergence training to the baseline measurements before vergence training for the CI subjects. Since the threshold used is the same for both pre and post-vergence training analysis, we assume that any potential differences observed within the data sets are mostly due to vergence training. The BOLD percent signal change for each ROI per subject comparing elevated activation observed during the vergence task to the baseline of sustained fixation was computed from the time series. The individual-subject percent signal change values were pooled to conduct the group-level statistics described below.

### Statistical analyses

The subject data were stratified into the following three groups: BNC, CI subjects before vergence training, and CI subjects after vergence training. An unpaired *t*-test was used to determine whether significant differences were observed between BNC and CI subjects before vergence training when analyzing (1) the peak velocity of convergence responses stimulated from 4° symmetrical convergence step stimuli and (2) the percent signal change of the BOLD fMRI signal within an ROI. A paired *t*-test determined whether the CI subjects exhibited significant changes comparing the pre and post vergence training measurements for the following parameters: (1) peak velocity of convergence responses stimulated from 4° symmetrical convergence steps, (2) percent signal change of the fMRI BOLD signal within an ROI, (3) CISS score, (4) NPC, (5) positive vergence amplitude ranges, and (6) near dissociated phoria. A linear regression analysis was conducted between the peak velocity of convergence responses stimulated from 4° symmetrical convergence steps and the BOLD percent signal change for the following ROIs: FEF, PPC, CV and Broca's region. Statistics were calculated using NCSS2004 (Kaysville, UT, USA). Significance was defined as a *p*-value < 0.05. Bonferroni correction for multiple parameters was not applied because of the limited number of subjects within the study. Figures were generated using MATLAB (Mathworks, MA).

## Results

### Convergence eye movements from symmetrical convergence step stimuli

**Figure 3:**
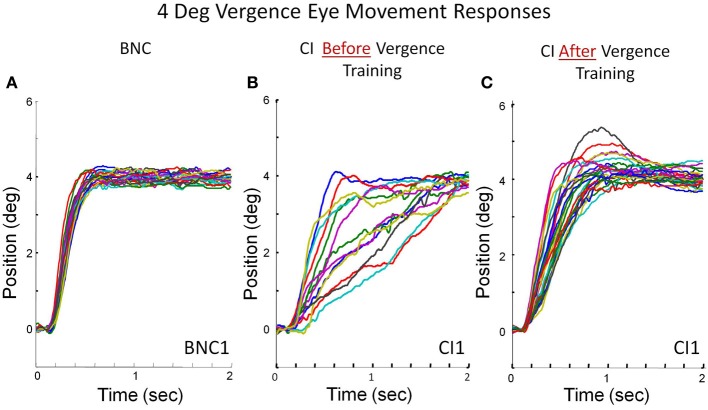
**Convergence eye movement responses stimulated from 4° symmetrical convergence step stimuli from a BNC (A), a CI subject before vergence training (B), and the same CI subject after 18 h of vergence training (C)**. Each colored trace is a single eye movement response denoted in degrees of rotation as a function of time (s).

Peak convergence velocity was one of the primary measurements within this study. Figure 3 plots multiple eye movements. Each colored line in Figure 3 is a convergence eye movement response evoked from a symmetrical 4° convergence step stimulus. Figure 3A is convergence responses from a BNC. The BNC subject attains fusion of the new target within the first half second. Figures 3B,C are from the same CI subject before and after vergence training, respectively. The CI subject before vergence training has more variability between responses compared to the BNC and can take up to 2 s to fuse the new target. After vergence training, the CI subject's responses are still slower than the BNC (comparing Figures 3A,C), but considerably faster than the subject's baseline responses (Figure 3B).

### Time-series of the BOLD signal from the ROIs studied

**Figure 4:**
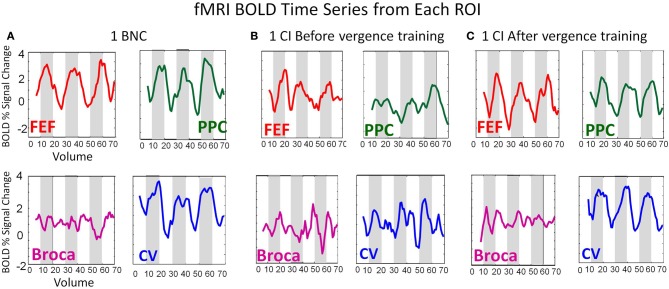
**BOLD time series from the FEF (red), PPC (green), Broca's Region (Purple), and CV (blue) from a BNC (A), one CI before vergence training (B), and the same CI after vergence training (C)**. The BOLD signal is plotted as the percent signal change as a function of volume. The time of repetition was 2 s. Hence, the time series lasts for a duration of 140 s.

Figure 4 shows data from two subjects, one BNC (Figure 4A), one CI subject before vergence training (Figure 4B), and the same CI subject after vergence training (Figure 4C). Figure 4 shows the average time series from the FEF-L (red lines), PPC-L (green lines), Broca-L (purple lines), and CV (blu shows data from two subjects, one e lines). Figure 4 plots the BOLD percent signal change as a function of volumes collected (70 volumes equating to 140 s in duration). The BNC shows an FEF time series which is more correlated (*r* = 0.66; *p* < 0.001) with the experimental block design (white and gray boxes for the 3.5 cycles of the experiment) compared to the CI subject before vergence training (*r* = 0.33; *p* < 0.01). After vergence training, this subject's FEF correlation with the block design increases (*r* = 0.73; *p* < 0.001). Similar trends are observed for the PPC and the CV. As expected, the time series from Broca's region (control ROI to study variability of a non-stimulated region) does not correlate with the experimental block design for the BNC and the CI before or after vergence training (*r* = 0.15 ± 0.05; *p* > 0.1).

### Group-level analyses

**Figure 5:**
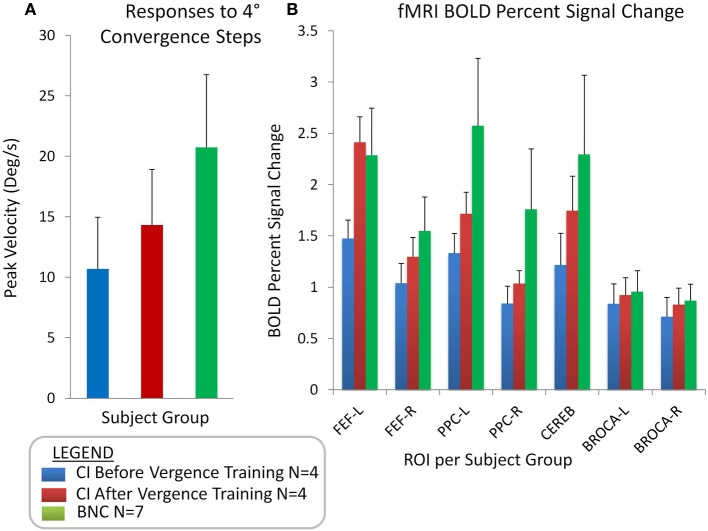
**Group-level analyses showing the average with one standard deviation of convergence peak velocities (°/s) evoked from 4° symmetrical convergence step stimuli (A) for CI subjects before vergence training (blue bar), CI subjects after vergence training (red bar), and BNC subjects (green bar)**. Group-level averages with one standard deviation for the BOLD percent signal change for the FEF-L, FEF-R, PPC-L, PPC-R, Cerebellar vermis, Broca-L, and Broca-R **(B)**.

The peak velocities elicited from 4° symmetrical convergence steps were averaged for the seven BNC and the four CI subjects before and after repetitive vergence training. Figure 5A plots the average (bar) with one standard deviation (error bar) of the peak velocity (°/s) from CI subjects before vergence training (blue bar), the same CI subjects after vergence training (red bar), and from BNC subjects (green bar). When comparing the BNC group with the CI before vergence training group, an unpaired *t*-test revealed that significant differences were observed (*T* = 2.92; *p* < 0.02). The CI subjects had significantly slower peak velocities evoked from 4° symmetrical convergence steps compared to BNC subjects. The CI subjects also exhibited significant changes in peak velocities to symmetrical 4° convergence steps when comparing the responses after vergence training to the baseline before vergence training responses, when using a paired *t*-test (*T* = 6.93; *p* < 0.02).

Figure 5B shows the average with one standard deviation for the group-level analysis of the percent change in the BOLD signal per ROI for the following groups: CI subjects before vergence training (blue bar), CI subjects after vergence training (red bar), and BNCs (green bar). When comparing the BNC to the CI data before vergence training using an unpaired *t*-test, significant differences were observed within the FEF, PPC, and CV (*t* > 2.3; *p* < 0.05). No significant differences were observed within Broca's region between the BNC and either the before or after vergence training CI datasets (*t* > 1.1; *p* > 0.3). A paired *t*-test showed the percent change in the BOLD signal in the FEF, PPC, and CV of the four CI subjects who participated in vergence training were significantly greater after training compared to the baseline values (*t* > 2.6; *p* < 0.001). No statistical difference was observed in Broca's region (*t* = 1.2; *p* > 0.3) when comparing the baseline and after vergence training data.

**Figure 6:**
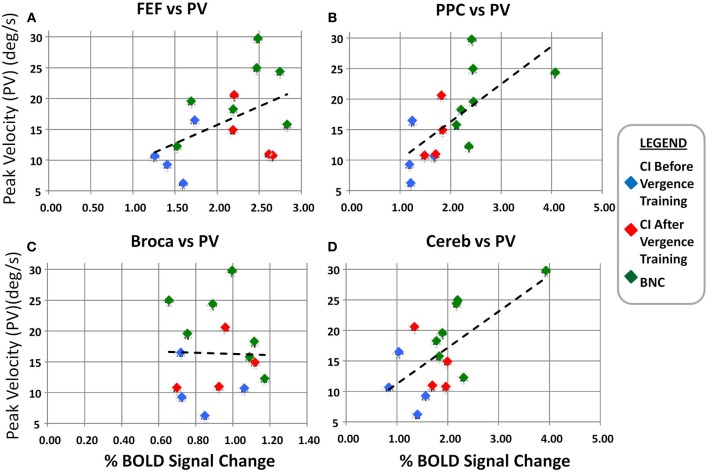
**Linear regression analysis of the convergence peak velocity (°/s) as a function of the percent BOLD signal change for the FEF (A), PPC (B), Broca's region (C), and Cerebellar Vermis (D)**. The left and right percent BOLD signal changes were averaged. The blue diamonds are from the CI subjects before vergence training, the red diamonds are the same CI subjects after vergence training, and the green diamonds are from the BNC subjects. The dashed black line is the linear regression. Significant correlation is observed between the convergence peak velocity from 4° symmetrical convergence steps and the BOLD percent signal change in the FEF, PPC, and Cerebellar Vermis.

A linear regression analysis was conducted of the average convergence peak velocity evoked from 4° symmetrical convergence steps as a function of the BOLD percent signal change for the FEF (Figure 6A), PPC (Figure 6B), Broca's Region (Figure 6C), and CV (Figure 6D). The left and right ROIs were averaged for Figure 6. A regression analysis revealed the convergence peak velocity of 4° symmetrical convergence steps from BNC and CI patients before and after vergence training was significantly correlated to the percent BOLD signal change within the FEF (*r* = 0.5; *p* < 0.05), PPC (*r* = 0.7; *p* < 0.01), and CV (*r* = 0.6; *p* < 0.01). Conversely, convergence peak velocity of 4° symmetrical convergence steps from BNC and CI patients before and after vergence training was not significantly correlated to the percent BOLD signal change within Broca's regions, which was the control ROI (*r* = −0.0059; *p* > 0.98).

### Clinical vision parameters

A paired *t*-test revealed a significant difference comparing the baseline (before vergence training) parameters and the after vergence training parameters for the following measurements: the NPC (*t* = 4.9; *p* = 0.04), BO positive fusional vergence range (*t* = 9.5; *p* = 0.01), near dissociated phoria (*t* = 11; *p* = 0.008), and CISS (*t* = 3.6; *p* = 0.05). All significant changes are improvements to each parameter studied.

## Discussion

The data support the hypotheses that were tested. Reduced convergence peak velocity from convergence step stimuli and functional activity within the FEF, PPC, and CV were observed in those with CI before repetitive vergence training compared to BNC subjects. Both convergence peak velocity and functional activity significantly improved after vergence training in the CI subjects when comparing the pre and post-vergence training measurements. The average peak velocity of convergence responses was significantly correlated to the BOLD percent signal change within the functional activity of the FEF, PPC, and CV neural substrates. The results of this study will be compared to those in the literature.

### Clinical implications of long-term adaptation evoked through vergence training

Understanding the relationship between the functional activity within the FEF, PPC, and CV and convergence eye movement responses has both basic science and clinical applications. Although the majority of humans perform vergence movements with ease, the dysfunction known as CI is reported to be present within 4.2–7.7% of the population (Hokoda, 1985; Scheiman et al., 1996; Porcar and Martinez-Palomera, 1997; Rouse et al., 1998, 1999). CI is an eye co-ordination and alignment problem, which can result in visual symptoms when engaged in reading or performing other near work (Scheiman et al., 2011).

The randomized clinical trial, the Convergence Insufficiency Treatment Trial (CITT), showed that OBVAT was successful in 73% of children, resulting in significantly improved symptoms, NPC and positive vergence amplitude (Scheiman et al., 2009). Clinical signs and symptoms were sustained 1 year post-therapy for most subjects (CITT, 2009). OBVAT is composed of symmetrical, horizontal convergence movements. Hence, although the stimulus used within this current study may not occur often in natural viewing conditions, it is the basis of therapeutic interventions to treat patients with CI (Cooper et al., 1998; Scheiman and Wick, 2008; Scheiman et al., 2011).

The Dual-Mode model of vergence describes vergence as a two component system composed of a fusion initiating and a fusion sustaining component (Hung et al., 1986; Lee et al., 2012). The transient fusion initiating component is modeled as a preprogrammed control system mainly contributing to the vergence system's speed but is not necessarily very accurate. The fusion sustaining component is feedback controlled and facilitates the accuracy of the vergence system. The present data suggest that the fusion initiating component, which mainly contributes to the vergence peak velocity, is modified after vergence training. The CI subjects had reduced peak velocity before training, which increased after training. The results of this study suggest that vergence training protocols may concentrate on the stimulation of the preprogrammed portion of vergence system.

Investigations identifying the neural substrates responsible for vergence oculomotor learning are scare. Several saccade oculomotor studies suggest the oculomotor vermis is responsible for oculomotor learning within the saccadic system (Iwamoto and Kaku, 2010; Prsa and Thier, 2011). Evidence also suggests that the FEF can be modified through adaptation when studying saccades (Lee et al., 2011). This present study provides a critical step in understanding the brain-behavior relationship of how vergence training is inducing changes to the functional activity within the FEF, PPC, and CV, which in part mediates convergence oculomotor responses. Future research can study neurological differences between various vergence training protocols to determine how changes within neural substrates facilitate an improvement in visual comfort while performing near work such as reading. Such knowledge could ultimately lead to an improvement in vergence training protocols.

### BOLD percent signal change in relation to other bodies of literature

Non-human primate single cell electrophysiology studies have investigated the influence of disparity in the FEF using symmetrical step stimuli (Gamlin and Yoon, 2000), near and far targets (Ferraina et al., 2000), and smooth sinusoidal tracking stimuli (Fukushima et al., 2002; Akao et al., 2005). The FEF and PPC have also been shown to be involved in predictive oculomotor learning (Tseng et al., 2013). The PPC encodes for different binocular distances defined by different vergence angles studying primates using single cell recordings (Genovesio and Ferraina, 2004; Ferraina et al., 2009; Breveglieri et al., 2012) and humans using transcranial magnetic stimulation (Kapoula et al., 2001, 2004, 2005; Yang and Kapoula, 2004) and fMRI (Quinlan and Culham, 2007; Alvarez et al., 2010a; Alkan et al., 2011a,b). Primate single cell studies have also shown that the CV is used to mediate a vergence response (Gamlin et al., 1996; Nitta et al., 2008a,b). Patients, particularly those with lesions to the cerebellar vermal regions, exhibit a decrease in slow tracking vergence (Sander et al., 2009).

This present study further confirms that the FEF, PPC, and CV are metabolically active during a vergence task. The novelty of this study's results is that the functional activity of the FEF, PPC and CV are: (1) reduced in CI subjects at baseline compared to BNC subjects, (2) significantly increased after 18 h of vergence training to levels more similar to those exhibited by the BNC subjects, and (3) significantly correlated to the convergence peak velocity elicited from 4° symmetrical convergence stimuli. The results support the hypothesis that subjects with CI have reduced functional activity within the vergence neural substrates and reduced peak velocity of convergence responses compared to BNC. Results further support that after vergence training; the functional activity improves to levels more similar to those observed in BNC subjects.

### Study limitations and future directions

The reduced strength in fMRI activity and convergence peak velocity measurements observed from CI subjects compared to BNC is recommended for investigation in a masked randomized clinical trial where both CI and BNC participate in vergence training. Such a study could determine whether these differences between BNC and CI subjects generalize in a larger population and hence may, in part, explain asthenopia in CI patients.

The techniques used within the present study could also be applied to study the brain-behavior relationships of other oculomotor and vision dysfunctions. For example, Bucci and colleagues studied vergence insufficiency patients whose symptoms included headache and vertigo before and after orthoptic vergence training (Bucci et al., 2004, 2006a,b, 2011; Jainta et al., 2011). The techniques presented here could be used to better understand the mechanisms underlying vergence training for those with other visual and vestibular dysfunctions.

## Conclusions

The data collected within this study support that CI subjects have significantly reduced convergence peak velocity to 4° symmetrical convergence steps and BOLD percent signal change within the FEF, PPC, and CV compared to BNC subjects. Both convergence peak velocity and BOLD percent signal changes within the FEF, PPC, and CV significantly improved post-vergence training in CI subjects compared to their baseline measurements. The convergence peak velocity was significantly correlated to the BOLD percent signal change in the FEF, PPC, and CV. Results suggest that repetitive vergence training leads to an increase in the functional activity of the FEF, PPC, and CV which may in part lead to the increase in convergence peak velocity to symmetrical step stimuli.

### Conflict of interest statement

The authors declare that the research was conducted in the absence of any commercial or financial relationships that could be construed as a potential conflict of interest.
